# Lymphangioleiomyomatosis Presenting as Recurrent Pneumothorax

**DOI:** 10.7759/cureus.11102

**Published:** 2020-10-23

**Authors:** Mihir Odak, Kajol Anandani, Patrick J Rogers

**Affiliations:** 1 Internal Medicine, Jersey Shore University Medical Center, Neptune City, USA; 2 Emergency Medicine, Jersey Shore University Medical Center, Neptune City, USA

**Keywords:** lymphangioleiomyomatosis, tuberous sclerosis, spontaneous pneumothorax, renal angiomyolipoma

## Abstract

Lymphangioleiomyomatosis (LAM) is a disorder that causes cystic disease in the lungs. This condition is associated with renal angiomyolipomas and commonly occurs in individuals with tuberous sclerosis. Despite its frequent association with tuberous sclerosis, LAM is a rare condition and is often underdiagnosed. An identification of the array of signs for LAM is necessary to start the patient on appropriate long-term guideline-directed medical therapy.

A 24-year-old female patient with a past medical history of tuberous sclerosis, gestational hypertension, stable renal angiomyolipoma, and recent pneumothorax presented to our emergency department complaining of four weeks of productive cough. On presentation, she was found to have stable vital signs, and on examination, she had absent breath sounds in the right basilar and anterior mid-lung field compared to the left. Subsequent imaging confirmed a recurrence of pneumothorax from her visit three months prior to her current presentation as well as progressive cystic lung disease and an unchanged angiomyolipoma, suggestive of LAM.

We present this article with the hope of raising the index of suspicion for LAM in the setting of the particular signs and symptoms and to encourage prompt stabilization of the patient and initiation of guideline-directed medical therapy and strict follow-up to provide the greatest possible improvement in the patient’s quality of life.

## Introduction

Lymphangioleiomyomatosis (LAM) is a cystic disorder that primarily affects the lungs of females of childbearing age [1]. It occurs as a result of mutations in tuberous sclerosis genes, resulting in cystic lung disease in concurrence with extrapulmonary angiomyolipomas, commonly found in the kidneys. LAM more frequently occurs in association with tuberous sclerosis complex (TSC), a condition characterized by hamartomatous lesions in the brain, heart, lungs, and liver and complicated by developmental delay and autism [1]; however, a rarer sporadic form can also occur. LAM is extremely rare, and its association with TSC has a prevalence of 26% [1]. We present a case of a female who presented with a pneumothorax, who was also found to have significant progressive cystic lung disease, in the hope of encouraging a higher index of suspicion for LAM when cystic lung disease and renal angiomyolipomas are seen on imaging studies, especially in the setting of a history of tuberous sclerosis.

## Case presentation

The patient is a 24-year-old African-American female with a past medical history of tuberous sclerosis, gestational hypertension, stable renal angiomyolipoma, and recent pneumothorax, most recently three months prior to admission. She presented with a four-week history of productive cough. The cough was described as occurring intermittently throughout the day, productive of light-yellow colored sputum, and being associated with rhinorrhea and sinus congestion. She reported her uncle as a sick contact, who was being treated for an upper respiratory infection at the time she developed symptoms. The patient endorsed shortness of breath with mild exertion. The patient also complained of increased urinary frequency and dysuria that had started three days prior to admission; however, she denied any complaints of headaches, fever, chest pain, weakness, changes in vision, nausea, vomiting, or changes in bowel habits. The patient admitted to actively smoking tobacco and smokes approximately five cigarettes daily.

On presentation to the emergency department, the patient was not in respiratory distress. Her vital signs showed a blood pressure of 148/77 mmHg, heart rate of 74 beats per minute, temperature of 98.3 degrees Fahrenheit, and oxygen saturation of 97% on room air. The patient’s physical examination was significant for absent breath sounds in the right basilar and anterior-mid lung field compared to the left with no wheezes or rales. Her examination was otherwise benign. Her laboratory studies are shown in Table 1.

**Table 1:** Laboratory values Bun, blood urea nitrogen; WBC, white blood cells

Laboratory	Value	Reference Value
Sodium	137 mmol/L	136–145 mmol/L
Potassium	4.1 mmol/L	3.5–5.2 mmol/L
Chloride	108 mmol/L	96–110 mmol/L
Bicarbonate	21 mmol/L	24–31 mmol/L
BUN	15 mg/dL	5–25 mg/dL
Creatinine	0.73 mg/dL	0.44–1.00 mg/dL
Glucose	97 mg/dL	70–99 mg/dL
Hemoglobin	14.0 g/dL	12.0–16.0 g/dL
WBC	9.1 k/uL	4.5–11.0 k/uL
Platelets	280 k/uL	140–450 k/uL
Urinalysis	Urine glucose: negative; urine ketones: negative; urine bilirubin: negative; urine specific gravity: 1.014; blood: negative; pH: 6.5; urine protein: negative; urine nitrites: negative; urine leukocytes: trace	Urine glucose: negative; urine ketones: negative; urine bilirubin: negative; urine specific gravity: 1.005–1.030; blood: negative; pH: 4–8; urine protein: negative; urine nitrites: negative; urine leukocytes: negative
Urine culture	No growth	No growth

**Figure 1:**
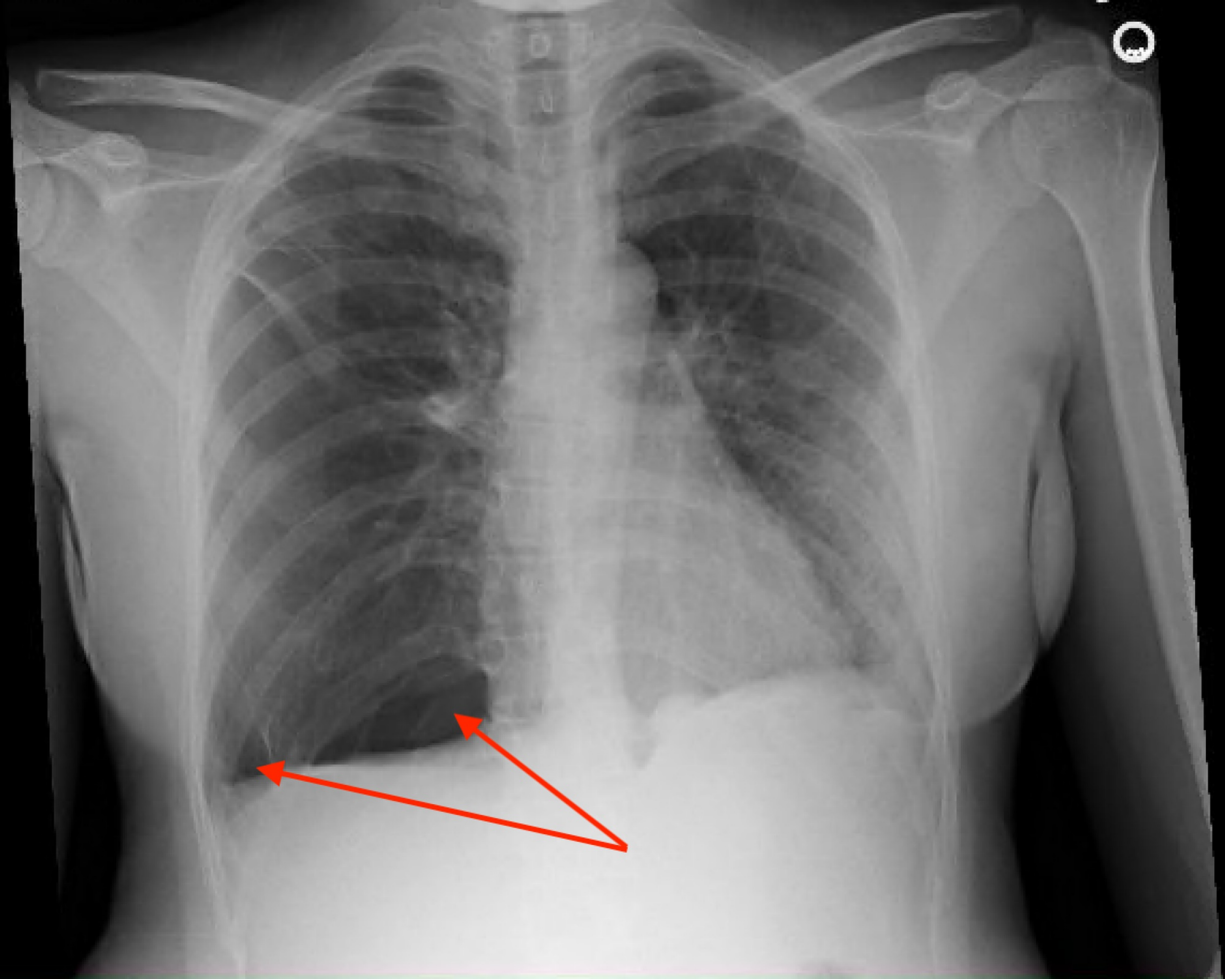
Chest X-ray showing right-sided basilar and lateral pneumothorax (red arrows).

**Figure 2:**
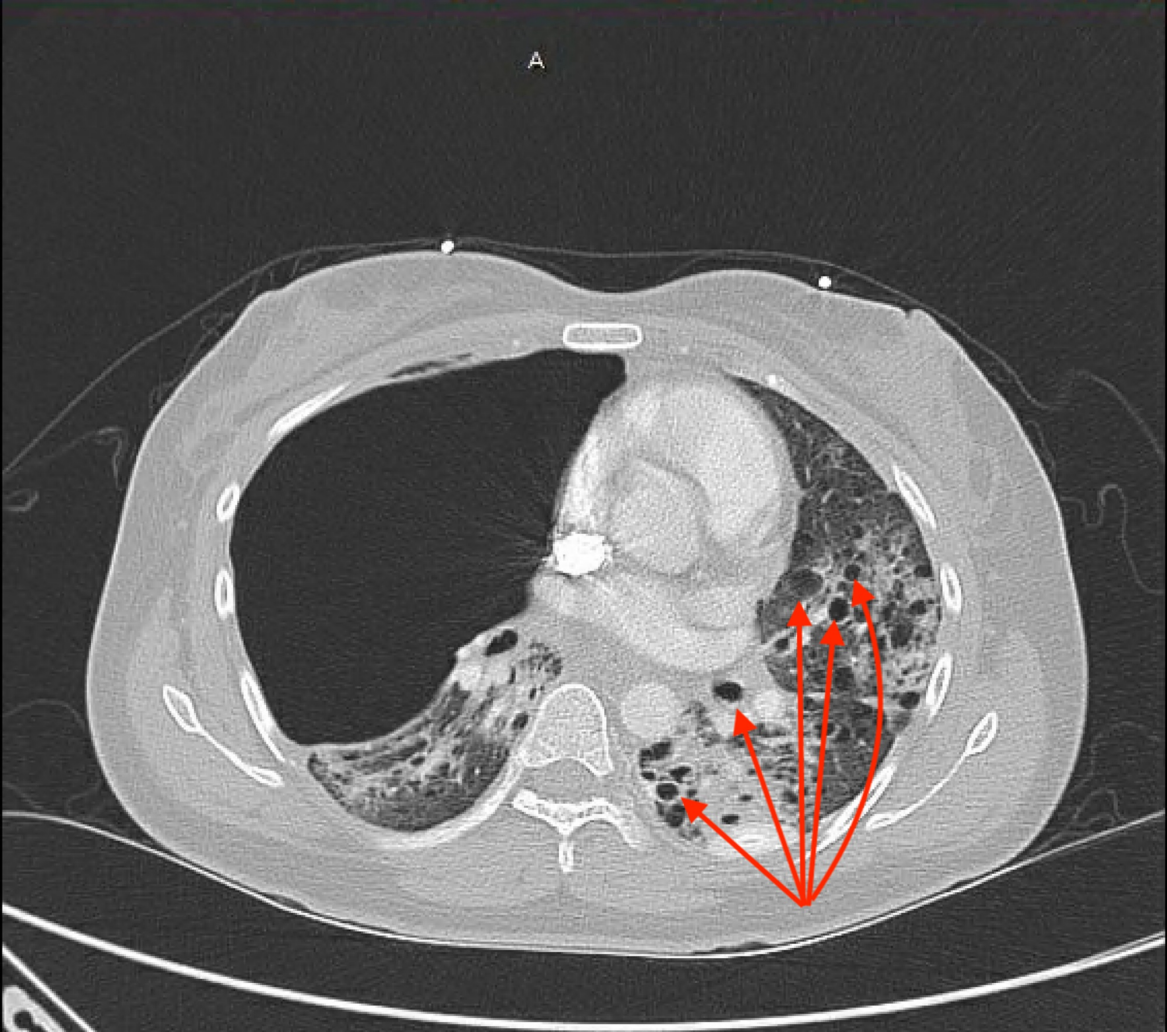
CT of the chest performed on follow-up visit showing multiple moderately thick-walled cysts located throughout the lung parenchyma with progression (red arrows).

**Figure 3:**
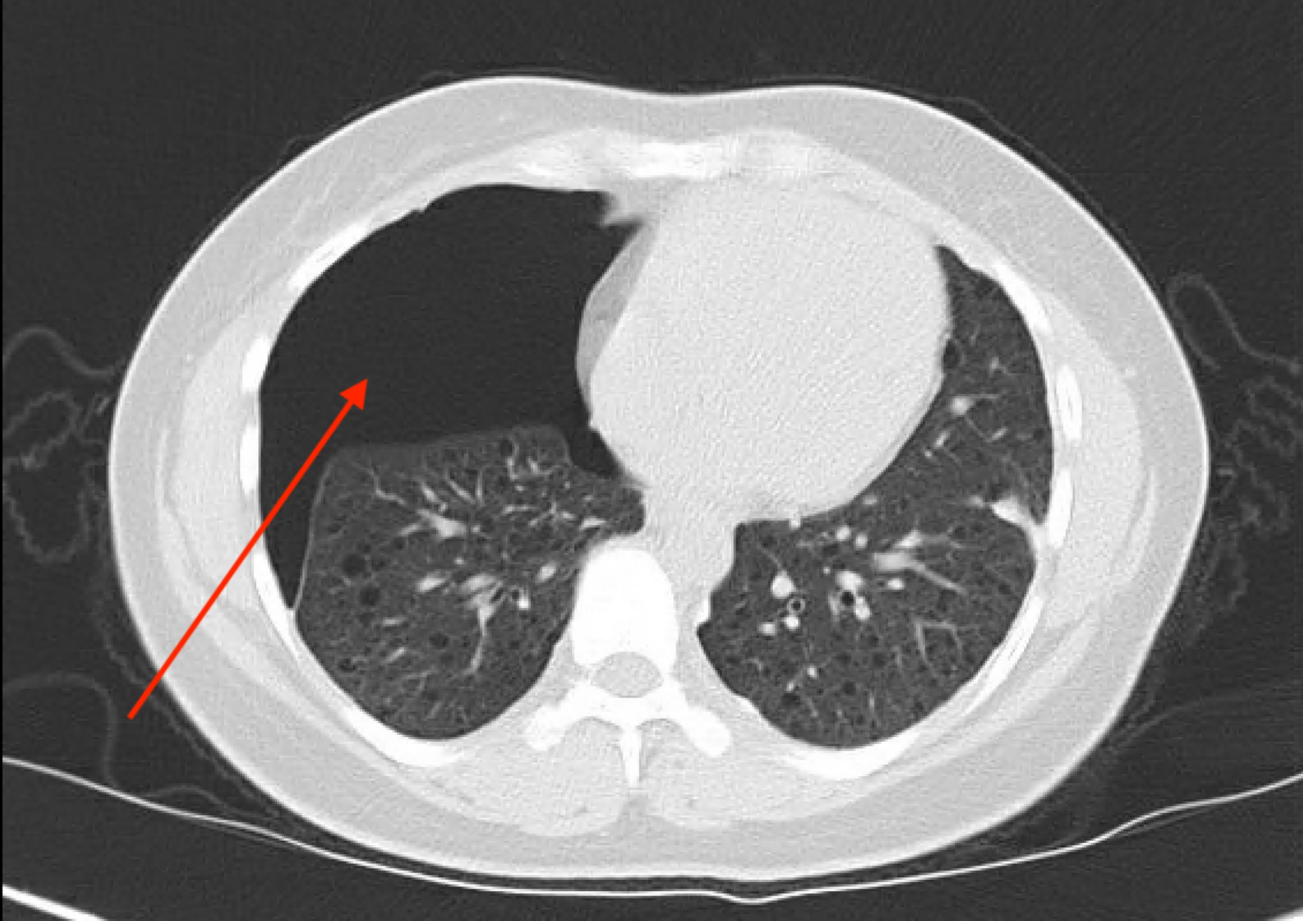
CT of the chest performed on initial visit showing a moderate-sized right anterior pneumothorax (red arrow).

**Figure 4:**
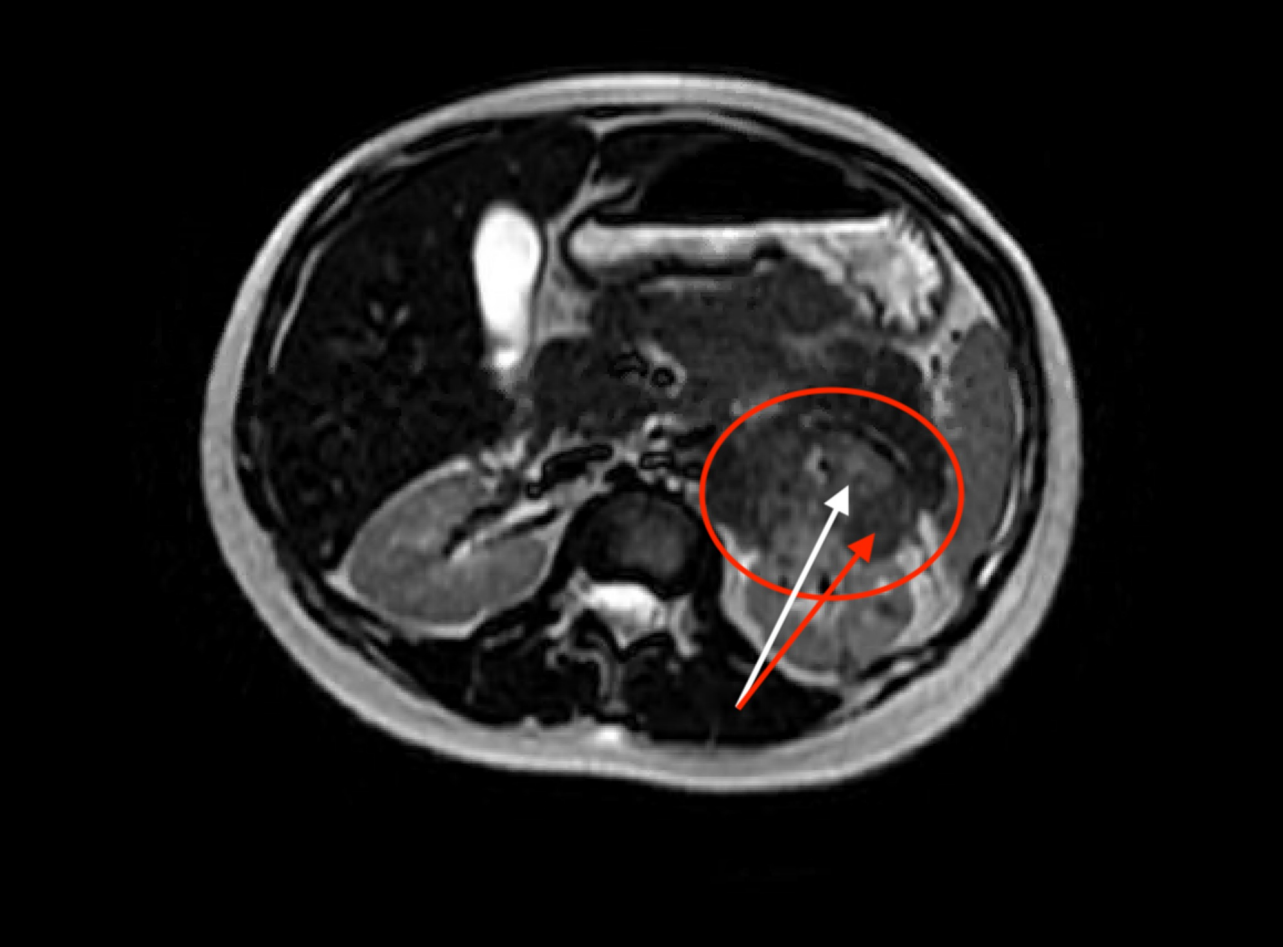
MRI of the abdomen without contrast showing a large heterogeneous mass arising from the superior aspect of the left kidney (red circle). Dense area (white arrow) contrasting with hypodense area (red arrow) suggesting the presence of fat and hypervascularity, indicative of angiomyolipoma.

She was evaluated further with a chest X-ray (Figure 1), computed tomography (CT) of the chest, abdomen, and pelvis (Figure 2), which was compared to her CT chest from her visit three months prior to her current admission (Figure 3), and magnetic resonance image (MRI) of the abdomen (Figure 4). The summary of imaging impressions is given in Table 2.

**Table 2:** Imaging

Imaging Study	Result
Chest X-ray	Right-sided basilar pneumothorax
CT of the chest	Large right-sided lung bulla and multiple moderately thick-walled cysts in the lung parenchyma
CT of the abdomen/pelvis	Large right basilar pneumothorax, heterogeneous-enhancing mass in the superior pole of the left kidney, suggestive of existing angiomyolipoma; multiple small cysts on the left kidney
MRI of the abdomen	Large heterogeneous mass arising from the superior aspect of the left kidney, likely stable angiomyolipoma

The patient was determined to have developed a recurrent spontaneous pneumothorax as a result of her cough. She received a dose of albuterol-ipratropium nebulizer therapy and a one-time 500 mg oral dose of levofloxacin followed by a chest tube placement. The patient’s pneumothorax ultimately resolved. Upon removal of the chest tube three days later, the patient was discharged to her home in stable condition, with advice to follow-up regularly with the medical team in the outpatient clinic.

## Discussion

The prevalence of TSC-associated LAM is 26% [1]. This low prevalence may be due to an underdiagnosis of LAM. The presence of tumors in the brain, heart, and kidneys, as is seen in tuberous sclerosis [2], may also lead practitioners to consider pneumothorax as a complication of lung tumors due to tuberous sclerosis rather than classifying it as LAM. The imaging study, however, may reveal no lung tumors, and cystic disease instead, as was the case for our patient. Although the patient population most frequently affected by this condition is females of reproductive age, post-menopausal women can also be affected [1]. The disease occurs as a result of mutations in the TSC genes, and it may also occur sporadically in a small percentage of the population. The pathophysiology of the condition involves a two-hit mutation hypothesis of the TSC1 and TSC2 genes [1]. As the TSC genes code for the proteins hamartin and tuberin, the absence or malfunction of these proteins contributes to the physical and radiological manifestations of LAM. Tuberin is responsible for cell growth and proliferation, whereas hamartin is thought to facilitate the reorganization of cytoskeletal elements; therefore, the dysfunction or absence of these proteins leads to focal loss of adhesions and the formation of cysts [3].

The most common presenting symptom for LAM is pneumothorax, and this is typically associated with dyspnea and chylous pleural effusions [4,5]. Patients also have extrapulmonary manifestations of LAM, particularly angiomyolipomas [5]. The diagnostic guidelines for LAM, as described in Johnson SR et al., include a high-resolution CT scan for characteristic cystic lesions in the lung and detection of extrapulmonary manifestations of LAM [6]. As LAM is frequently associated with TSC, additional imaging studies may reveal tumorous lesions in the brain and heart. If these findings are positive, a pathology study is not necessary, and the patient may be treated accordingly for LAM [6]. The treatment involves targeted management of each of the manifestations. This includes pleurodesis for pneumothorax and thoracocentesis for effusions [6]. For small (<4 cm) asymptomatic angiomyolipomas, current recommendations are to not treat but follow-up yearly with ultrasound or CT scan. For symptomatic angiomyolipomas, conservative management is preferred, which is escalated to embolization for actively bleeding patients and nephron-sparing surgery for potentially malignant lesions [3].

Given this condition’s rarity, a diagnosis of LAM was not suspected for our patient on her initial presentation. The recurrence of pneumothorax on her subsequent presentation, along with her progressive cystic lung appearance on CT scan, history of stable angiomyolipoma, and tuberous sclerosis, led our medical team to make the diagnosis of LAM. The American Thoracic Society guidelines on the diagnosis of LAM recommend a high-resolution chest CT scan to evaluate for cystic lung disease in cases of suspected LAM. If positive, this can be followed by a correlation with positive personal or family history of TSC. If there is a positive personal or family history of TSC, the diagnosis of LAM can be confirmed at this point. If negative, a non-contrast CT scan or MRI of the abdomen may be obtained, and visualization of renal angiomyolipomas can serve as a confirmation of LAM [7]. The recommendations also include performing pleurodesis when patients initially present with pneumothorax rather than on subsequent presentations with pneumothorax [7]. Our patient was treated with a chest tube placement, which resolved her pneumothorax. The patient was then discharged with a plan in place for close follow-up.

It is therefore important to have a high index of suspicion for LAM, as the management involves resolving the pneumothorax, providing long-term breathing treatments, treatment of angiomyolipomas, close follow-up of the patient, and cessation of smoking tobacco. Spontaneous pneumothorax carries an annual incidence of eight cases out of 100,000, with tobacco smoking adding an eight times higher risk [8]. Our patient greatly benefited from our high index of suspicion and received prompt and targeted guideline-directed medical therapy for newly diagnosed LAM in the setting of tuberous sclerosis.

## Conclusions

TSC-associated LAM is a rare but potentially debilitating illness that can significantly impact patients’ way of living. It is a disease that requires close monitoring and frequent follow-up, and for this reason, it is an important inclusion in a list of differential diagnoses when a patient presents with recurrent pneumothorax in the setting of angiomyolipoma. By way of this article, we hope to encourage a greater degree of suspicion for tuberous sclerosis-associated LAM, as the prompt diagnoses of it can set the patient on a treatment course that will bring them relief and an enhanced quality of life.
